# Regional Anesthesia With Pectoral Blocks as a Non-inferior Approach for Mastectomy Analgesia: A Quality Improvement Study

**DOI:** 10.7759/cureus.73086

**Published:** 2024-11-05

**Authors:** Holden Lewis, J. Holt Evans, Michael Mullen, Andrew Gustetic, Seiha Kim, Miles Lane, Rebecca Jablonski

**Affiliations:** 1 Medicine, Edward Via College of Osteopathic Medicine, Spartanburg, USA; 2 Anesthesiology, Spartanburg Regional Healthcare System, Spartanburg, USA; 3 Statistics, Spartanburg Regional Healthcare System, Spartanburg, USA; 4 Nursing, Spartanburg Regional Healthcare System, Spartanburg, USA

**Keywords:** average length of stay, mastectomy, pectoral nerve block, perioperative pain management, quality improvement and patient safety

## Abstract

Introduction

Breast cancer is one of the most common female malignancies in the United States and often necessitates surgical interventions that carry a substantial risk of postoperative pain. Pectoral nerve blocks have emerged as a simpler alternative for providing regional perioperative analgesia to the chest wall in breast cancer surgery. This retrospective study evaluated the impact of implementing a novel regional anesthesia protocol centering on the use of pectoral nerve blocks for patients undergoing radical mastectomy at a small regional hospital in Spartanburg, South Carolina.

Methods

A retrospective study was conducted to examine the effects of peripheral nerve blocks, specifically pectoral nerve blocks, on intra- and postoperative milligram morphine equivalent consumption and postoperative length of stay for 168 mastectomy patients at Spartanburg Medical Center between June 2022 and June 2023. The association between anesthesia regimen received, length of stay, and perioperative milligram morphine equivalents consumed was examined using Wilcoxon rank sum testing.

Results

Patients who received pectoral nerve blocks (n = 23) demonstrated a 31.53% decrease in milligram morphine equivalent consumption in comparison to patients who received other types of peripheral nerve blocks within the same perioperative window. The length of stay for study patients who received pectoral nerve blocks (1.07 days) was grossly comparable to that for patients who received any other type of regional nerve block for their mastectomy over the course of the investigation (0.92 days).

Conclusions

For the provision of regional analgesia for mastectomy, pectoral nerve blocks were demonstrated to be non-inferior to other types of peripheral blocks traditionally used in this setting. After the change in protocol to pectoral nerve blocks in January 2023, mastectomy patients receiving pectoral nerve blocks required less perioperative pain medication, with no significant adverse impact on length of stay.

## Introduction

Problem description and available knowledge

Breast cancer is the second most common cancer among women in the United States, and it is estimated that approximately one in eight US women will develop breast cancer at some point in her life [1,2]. Surgery constitutes a significant therapeutic intervention in the management of breast cancer, and patients undergoing procedures like mastectomy are at risk of experiencing significant postoperative pain that can cause chronic discomfort and restriction of motion if left untreated [3-5].

Thoracic paravertebral blocks (TPVBs) are widely used to achieve perioperative analgesia for breast cancer patients undergoing radical mastectomy; however, they are demonstrated to be technically demanding and confer the risk of complications such as pneumothorax [6]. Another regional anesthesia regimen widely used in the setting of mastectomy is the serratus anterior plane block (SAPB). While this modality blocks the second through sixth intercostal nerves, it does not provide coverage to the median and lateral pectoral nerves (PECS), both of which face the risk of injury during mastectomy [7,8]. Numerous studies have demonstrated the safety and efficacy of PECS blocks as a less technically demanding alternative to TPVB and SAPB for patients undergoing breast cancer surgeries [9,10]. PECS blocks are efficacious due to their provision of comprehensive analgesic coverage that includes the PECS [7]. There are two types of PECS blocks known colloquially as “PECS I” and “PECS II.” PECS I blocks cover only the lateral and medial PECS, whereas PECS II blocks cover the long thoracic nerve, thoracic intercostal nerves from T2 to T6, and thoracodorsal nerve in addition to the two PECS [7]. Comprehensive regional anesthesia plans utilizing PECS blocks for mastectomy procedures typically include both PECS I and PECS II blocks [11]. Compared to other blocks used in this setting, PECS II blocks have been associated with significantly lower 24-hour postoperative patient pain scores, lower postoperative opioid consumption, and a decreased incidence of chronic pain after surgery [12].

Rationale

One of the principal drivers behind this investigation was to determine whether a shift in a regional anesthesia protocol informed by the quality improvement cycle, as outlined by the Agency for Healthcare Research and Quality, could yield a significant improvement in perioperative outcomes for patients receiving mastectomies [13]. According to the literature, roughly 10% of patients prescribed opioids after surgery develop an opioid use disorder, and receiving opioids for pain management in the surgical setting is a risk factor for the development of prolonged opioid use even among opioid-naive patients. Using the quality improvement cycle as a guide, the anesthesiology department at Spartanburg Medical Center, a regional hospital and tertiary care center in Spartanburg, SC, and part of the Spartanburg Regional Healthcare System (SRHS) network, wanted to gather data on possible solutions to reduce perioperative opioid consumption for patients receiving mastectomies. A review of existing literature indicated that reducing morphine consumption postoperatively can reduce patient risk of addiction and that the additional coverage offered by PECS blocks could potentially decrease perioperative opioid consumption [12,14]. When taken together, these factors indicated that the regional anesthesia protocol for mastectomies at Spartanburg Medical Center could be improved by transitioning from using predominately SAPB or erector spinae blocks (ESPB) to using PECS blocks as the first-line agent for perioperative analgesia. The department began this transition in September 2022 [15].

Specific aims

In this study, the exploration of the non-inferiority of PECS blocks compared to all other forms of regional blocks was explored. The primary and secondary outcomes analyzed were intra- and postoperative milligram morphine equivalents (MME) consumed and patient length of stay (LOS) from admission to discharge, respectively.

## Materials and methods

Context

This study was initiated to investigate the impact of transitioning the Spartanburg Regional Anesthesia Department's standard regional analgesia approach for mastectomy patients from ESPB to PECS blocks. The shift aimed to assess the influence on perioperative MME consumption and LOS. The underlying motivation was to align with a quality improvement cycle, recognizing the importance of reducing postoperative opioid consumption to mitigate the risk of opioid use disorder.

Intervention

The intervention consisted of administering PECS blocks as the primary regional analgesia method for mastectomy patients, following a standardized protocol at SRHS, with all patients receiving a balanced general anesthetic per local protocol. The team responsible for the intervention included anesthesia providers and healthcare professionals involved in perioperative care. To study the intervention's impact, a retrospective observational study compared two distinct time periods based on the department’s transition to PECS blocks: June 2022-December 2022 and January 2023-June 2023. The study aimed to establish non-inferiority by assessing the temporal association between the introduction of PECS blocks and changes in perioperative MME consumption and LOS. Perioperative and postoperative MME consumption and LOS were measured to reduce the risk of an opioid use disorder, with operational definitions consistent with medical standards. Data completeness and accuracy were ensured through chart abstraction by the quality department of SRHS.

Measures

Both qualitative and quantitative methods were used for data analysis. Quantitative methods involved statistical comparisons of MME consumption and LOS between the two time periods, while qualitative methods considered contextual factors and other various inclusion and exclusion criteria influencing outcomes. The inclusion criteria consisted of patients who were at least 18 years old, had `no contraindications to nerve blocks, and had no allergies to local anesthetics or adjuvants. The exclusion criteria consisted of patients with allergies or contraindications to nerve blocks, local anesthetics, or adjuvants and patients who refused a peripheral nerve block. One of the measures applied was the Wilcoxon rank sum test. This test allows for the comparison of two independent data sets when those sets do not meet the criteria of normality for standard parametric tests. This test allows the assessment of whether there was a significant difference in the distribution of ranks between the two sets, providing insights into the effectiveness of the intervention in reducing opioid consumption and length of hospital stay. The analysis also included ongoing assessment of contextual elements contributing to success, failure, efficiency, and cost through regular evaluations of intervention implementation.

Ethical considerations

Ethical aspects were addressed by obtaining approval from the Spartanburg Regional Medical Center Office of Research Compliance, classifying the study as a quality improvement initiative not requiring institutional review board (IRB) review. Potential biases from the retrospective nature of the study and data input processes were acknowledged. The study prioritized transparency and integrity, providing valuable insights into the effectiveness of PECS blocks while recognizing its limitations. The authors used the Revised Standards for Quality Improvement Reporting Excellence (SQUIRE 2.0) guidelines in reporting this study.

## Results

Among the cohort of 168 patients, 23 individuals (13.7%) received a PECS peripheral block, encompassing both PECS I and PECS II, while the remaining 145 (86.3%) patients underwent a different type of block (SAPB, TPVB, ESPB, etc.).

**Figure 1:**
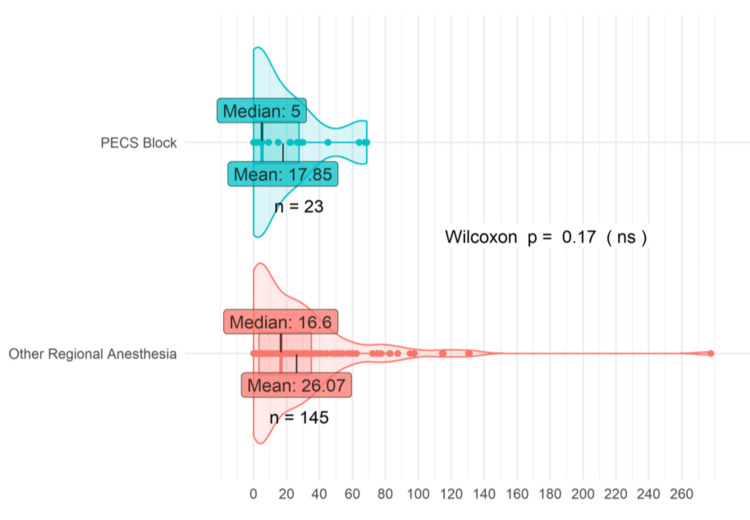
PECS blocks vs. other regional anesthesia MME. PECS blocks encompass PECS I and II. Other regional anesthesia encompasses all other peripheral nerve blocks including SAPB, TPVB, and ESPB PECS: pectoral nerve; MME: milligram morphine equivalent; SAPB: serratus anterior plane block; TPVB: thoracic paravertebral block; ESPB: erector spinae block

Figure 1 provides a visual approximation of the total MME consumed by patients who underwent a PECS block versus those who received other forms of regional anesthesia. The “other regional anesthesia” cohort included individuals who underwent a different type of peripheral nerve block, such as TPVP, ESPB, and SAPB. Total MME was derived by averaging the intraoperative MME, postoperative MME at 24 hours, and postoperative MME at 48 hours. The “PECS block” group comprised 23 patients, while the “other regional anesthesia” group consisted of 145 patients. In the “PECS block” group, both the median (five) and mean (17.85) total MME were observed to be lower than those of the “other regional anesthesia” group, which conferred a median (16.6) and mean (26.07) total MME. Patients who received a PECS block consumed 31.53% less MME perioperatively on average than patients who received another type of peripheral block. Additionally, the median MME consumed by patients who received a PECS block for their mastectomy was found to be 69.88% lower than for patients who received other peripheral blocks.

**Figure 2:**
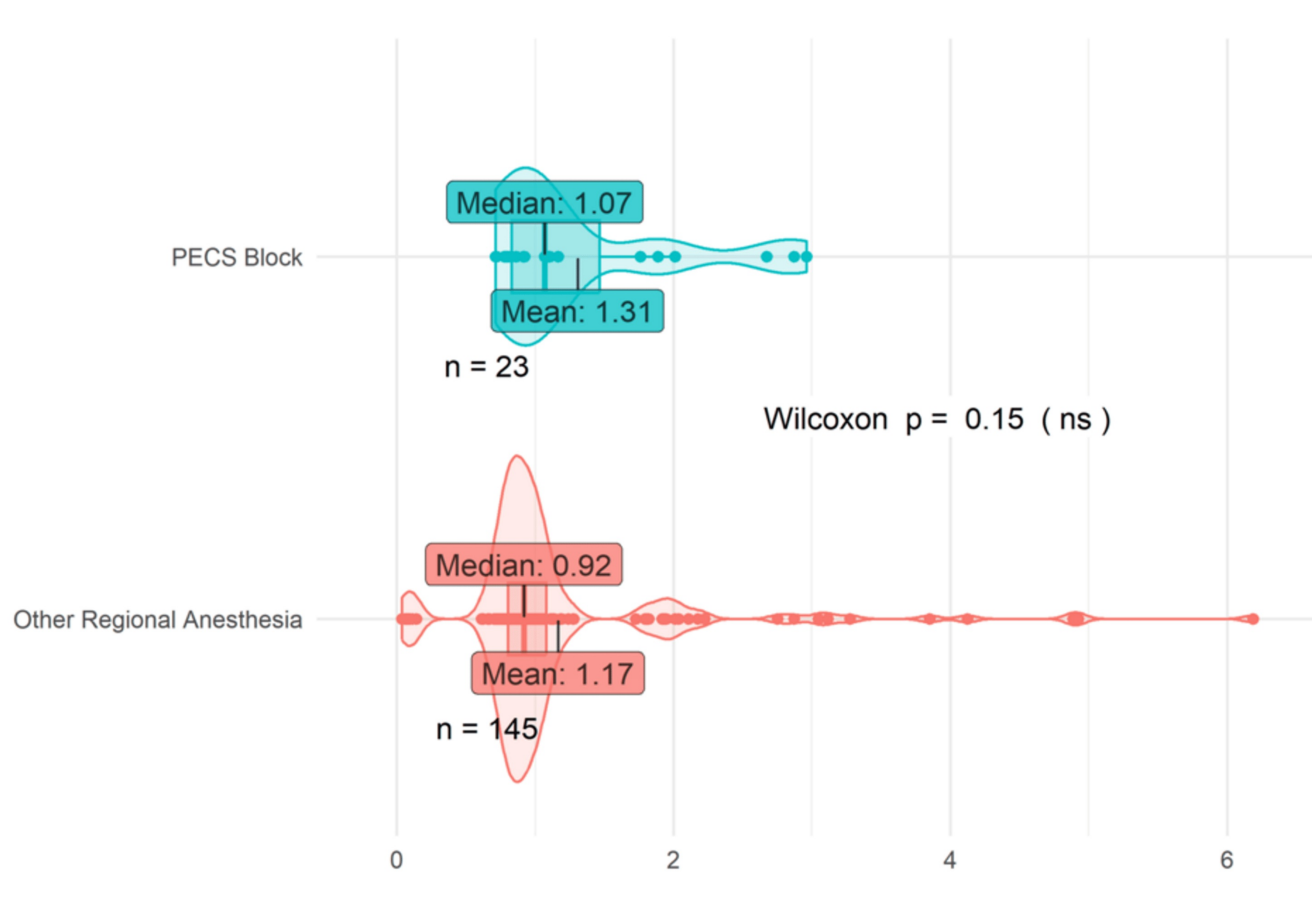
PECS blocks vs. other regional anesthesia LOS. PECS blocks encompass PECS I and II. Other regional anesthesia encompasses all other peripheral nerve blocks including SAPB, TPVB, and ESPB PECS: pectoral nerve; SAPB: serratus anterior plane block; TPVB: thoracic paravertebral block; ESPB: erector spinae block; LOS: length of stay

Figure 2 shows that the mean LOS for mastectomy patients who received PECS blocks was 1.31 days, while patients receiving an alternate peripheral block had a mean LOS of 1.17, which represented an 11.97% increase for the PECS cohort. The LOS value corresponds to the duration, in days, that patients spent in the hospital from admission to discharge. Patients who received PECS blocks also exhibited a slightly longer median LOS than those in the “other regional anesthesia” group: 1.07 days to 0.92 days, respectively. These observed differences were not found to be statistically significant via the Wilcoxon rank sum test, likely due to the sample size limitations of the PECS group (n = 23), especially when compared to the “other regional anesthesia” group (n = 145).

## Discussion

Summary

In this study involving patients at a regional hospital undergoing mastectomy procedures, PECS blocks were demonstrated to be non-inferior in terms of regional analgesia provision and impact on patient LOS. Adopting PECS blocks as the gold standard for regional anesthesia delivery proved a simple and effective way to enhance outcomes after surgery for patients undergoing various mastectomy procedures. Patients who received regional anesthesia for mastectomy following the department’s switch to focusing on PECS blocks in January 2023 required less pain medication perioperatively than those treated prior to the transition. Additionally, this investigation revealed that the change in anesthesia protocol did not significantly impact patient LOS within the post-anesthesia care unit (PACU).

The strengths of this manuscript include its relevance and applicability to the field of mastectomy analgesia. These findings pointing toward the non-inferiority of PECS blocks are synergistic with literature findings indicating their ease of use, facilitating their ready integration into practice. In addition, this study provides an example of how PECS blocks can be integrated quickly and efficaciously into an anesthesia protocol at a small regional hospital and indicates how similar results could likely be achieved at larger metropolitan and academic centers.

Interpretation

PECS blocks constitute a cutting-edge approach to regional anesthesia for surgeries involving the chest wall. Historically, the prevailing standard of care for such procedures revolved around alternative peripheral blocks, including TPVB, SAPB, and ESPB, all of which rely on blocking the long thoracic nerve to induce regional analgesia perioperatively [16]. PECS blocks distinguish themselves from these older techniques by impairing synaptic transmission within the medial and lateral PECS, in addition to the long thoracic nerve (PhD dissertation: Drake AJ. Mitigating Opioid Use Disorder and the Opioid Epidemic in the United States; 2023). This investigation demonstrated the aptitude of PECS blocks for the provision of comprehensive analgesia for various mastectomy procedures within a community hospital setting. The additional coverage provided by these blocks served as the likely driver of the benefits observed in this study.

In light of the escalating prevalence of the opioid epidemic across the United States, it has become increasingly imperative to employ precise and effective regional anesthesia for surgeries associated with a higher risk of nerve damage or persistent postoperative pain. The rise in annual opioid deaths in the United States to above 80,000 in 2021 underscores the pressing urgency of leveraging regional anesthesia in a way that can minimize perioperative opioid consumption, particularly for significantly invasive surgical interventions such as mastectomies [17]. Regional anesthesia holds the potential to curtail intraoperative and postoperative demand for opioids, which could reduce rates of opioid abuse following surgery. The benefits of regional anesthesia incorporating PECS blocks on perioperative opioid consumption were exemplified by the findings of this study. In addition, this decrease in acute pain has been seen with a decrease in chronic pain as well. Current literature illustrates how patients receiving a PECS block have experienced less chronic pain at 3-12 months postmastectomy, with only 14.9% of patients who received PECS blocks reporting chronic pain compared to 31.8% in patients who received general anesthesia [9]. The acute pain reduction found in this study, along with the current literature demonstrating the efficacy of PECS blocks for chronic pain, makes this regional block a viable anesthetic protocol for mastectomy procedures.

As the field of medicine advances through an array of quality improvement investigations, the incorporation of novel and efficacious regional anesthesia techniques assumes ever-increasing significance. PECS blocks, a technique that has been substantiated as a straightforward alternative to other more demanding or risky blocks for mastectomies, enable physicians to administer analgesia to the chest wall preoperatively with minimal or no sedation required (PhD dissertation: Drake AJ, 2023). Structuring regional anesthesia protocols around PECS blocks, validated for their efficacy and non-inferiority in comparison to other regional blocks, presents a compelling opportunity for both regional and larger metropolitan hospitals to provide more comprehensive care to patients undergoing mastectomies.

The primary motivation behind this study was to enhance the quality of regional anesthesia care for mastectomy patients at Spartanburg Medical Center. Given the ever-evolving landscape of medical literature, quality improvement studies such as this one are essential for the continued implementation of novel treatment measures that ensure the highest possible level of patient care. This study began by employing the widely accepted plan-do-study-act (PDSA) cycle, a common approach to quality improvement in healthcare [18]. The anticipated benefits of this approach were demonstrated in a smaller community hospital, resulting in reductions in MME and a negligible variance in LOS for patients undergoing mastectomy.

Limitations

While there are many strengths of this quality improvement initiative, some drawbacks exist. For instance, the small sample of patients who received PECS blocks during the study period (n = 23) limited the statistical significance of the study findings. Spartanburg Medical Center serves a largely rural patient population and likely receives a lower case volume than more urban tertiary care facilities. The hospital also lacks the expanded capacity for inpatient surgery found at larger centers. All these factors likely contributed to the limited sample size of this investigation. Despite these limitations, this study provides valuable insight into the effectiveness of PECS blocks in limiting the amount of MME consumed perioperatively by patients receiving mastectomies.

## Conclusions

In conclusion, PECS blocks emerge as a novel and non-inferior anesthetic strategy for patients undergoing mastectomy procedures. These blocks curb patient opioid consumption both perioperatively and within the PACU and underscore their non-inferiority by yielding nearly identical hospital LOS postprocedure to other blocks. Considering the opioid epidemic's gravity, the reduction of opioid usage during surgical interventions is of paramount importance to public health in the United States. This quality improvement initiative offers a compelling illustration of how an impactful change implemented within a small regional hospital can serve as a template for replication across larger metropolitan medical settings, thereby reinforcing prospects for enhanced patient care. Future studies could focus on the impact of implementing a PECS-centered regional anesthesia protocol in a larger setting with a diverse patient population and whether results such as these are replicable. Further studies could also delve into potential variance in efficacy between PECS I vs PECS II blocks for similar procedures.
